# Low Altruism as a Cause of Ostracism

**DOI:** 10.3389/fpsyg.2021.653217

**Published:** 2021-07-15

**Authors:** Lianqiong Huang, Yingge Zhu, Denghao Zhang

**Affiliations:** Department of Psychology, Faculty of Science and Technology, Renmin University of China, Beijing, China

**Keywords:** ostracism, altruism, social responsibility, empathy, moderated mediation model

## Abstract

This study focused on the causes of ostracism and explored the relationship between altruistic personality traits and ostracism. Using a combination of questionnaire surveys and laboratory experiments, results showed that: individuals with lower altruism were more vulnerable to be ostracized than those with higher altruism (Study 1 and Study 2). The relationship between altruism and ostracism was partially mediated by social responsibility (Study 3). When facing a low-altruistic target, the source would infer that the social responsibility level of this target was also low, thereby leading the ostracism intention to the target. Empathy did not moderate the relationship between altruism and ostracism. On the one hand, empathy did not moderate the direct effect of altruism on ostracism (Study 2); on the other hand, it also did not moderate the mediating role of social responsibility (Study 3). The strengths and limitations of this research are also discussed.

## Introduction

Ostracism, which refers to being excluded and ignored by others, is common and pervasive. Although it can serve a social function (Hales et al., 2016), it is painful for those who are being ostracized (Eisenberger et al., 2003). Ostracism negatively affects individuals and threatens their basic psychological needs (Williams, 2007), thus causing serious harm to individuals' physical and mental health (Williams and Nida, 2011). In response to ostracism, people often engage in aggressive behaviors against those who have ostracized them but sometimes even against innocent bystanders (DeWall et al., 2009).

In view of the universality of ostracism and its negative effects, previous researchers mainly focused on the results of ostracism (Riva et al., 2014; Nezlek et al., 2015) and how to deal with the effects (Williams and Nida, 2011), and relatively few studies focused on the causes of ostracism. However, only when we identify the causes of ostracism can we have a deeper understanding of it and develop more targeted interventions to reduce or eliminate the negative influences of ostracism. Therefore, exploring the effects of ostracism is not enough; its causes should also be studied.

## Altruism and Ostracism

According to the victim precipitation theory, individuals engaging in aversive behaviors do not randomly choose their targets; rather, individuals possessing certain characteristics are more likely to be targeted than others (Curtis, 1974; Olweus, 1978; Elias, 1986). Researchers have found that some personality characteristics of the ostracized are important reasons for their ostracism (e.g., Hales et al., 2016). In addition, some inappropriate characteristics or behaviors (such as aggression or antisociality, passivity and sensitivity, or arrogance and bossiness) displayed by individuals are also found to make them potential objects of workplace ostracism (Aquino and Bradfield, 2000; Aquino and Thau, 2009).

Altruistic personality traits refer to individual differences such as generosity, helping, and kindness that broadly affect altruistic behaviors (Rushton et al., 1981). Individuals low in altruism are less willing to help others and tend to be selfish and self-centered, and highly altruistic individuals are more willing and likely to help others (Luo and Dai, 2015). According to the evolutionary function of ostracism, humans have evolved a set of behavioral adaptation patterns to ostracize individuals who lack social skills, and this evolutionary function of ostracism may be one of the few benefits it can bring (Gruter and Masters, 1986; Kurzban and Leary, 2001). This suggests that a group (at least to some extent) prefers cooperative individuals over those who are burdensome. Previous studies have shown that when the ability of a group member is below average, such as an individual who tosses the ball slowly in an online ball-tossing game, he will be considered as a drag for the whole group and will be ostracized (Wesselmann et al., 2015). In the Prisoner's Dilemma, one way to promote teamwork is ostracism, where players who defect are expelled (Hirshlifer and Rassmusen, 1989).

In addition, biological theories on altruistic behaviors suggest that altruism can increase the adaptability of other individuals and maximize the replication and preservation of genes of the group to which the individual belongs (Wilson, 1976). In other words, altruistic behaviors can improve the adaptability of a group. As a comprehensive psychological quality, personality has a direct impact on individual behaviors (Zheng and Gu, 2012); thus, we speculate that individuals with low altruism may have fewer altruistic behaviors and are therefore more likely to be ostracized. Furthermore, altruism is a facet trait of the agreeableness dimension in the Big Five personality model; Hales et al. (2016) found that people are more willing to ostracize disagreeable targets than agreeable targets. Therefore, we propose:

**Hypothesis 1:** Altruistic personality would be negatively correlated with ostracism.

### Mediating Role of Social Responsibility

Why are targets with low altruistic personality traits more vulnerable to being ostracized? We predict that this may be because the ostracizers assess and infer their social responsibility. On the one hand, many studies have shown that there is a significant positive correlation between altruism and social responsibility (Staub, 1974; Peterson, 1983; Oliner and Oliner, 1988; Bierhoff et al., 1991). Maruyama et al. (1982) examined the relationship between social responsibility and altruistic behaviors of children who asked for a candy on Halloween and found that individuals' social responsibility increased the likelihood of contribution and the amount of candy donated. Although these studies focus on altruistic behaviors and social responsibility, they have shown that altruistic personality traits and behaviors are positively correlated. Highly altruistic individuals are more willing to share information in an organization (Lee et al., 2011) and are more inclined to hold the idea that altruistic behaviors need to pay more but get rewarded less (Kerber, 2010).

On the other hand, a large number of empirical studies show evidence of the significant negative correlation between social responsibility and ostracism. For example, Jin (2018) found a significant negative correlation between conscientiousness and workplace bullying. Yan (2011) also found that conscientiousness, extraversion, agreeableness, and openness are negatively correlated with workplace ostracism. Because social responsibility consists of conscientiousness (Luo and Dai, 2015), we speculated that social responsibility may play a mediating role in the effect of altruism on ostracism. Therefore, we propose:

**Hypothesis 2:** Social responsibility plays a mediating role in the relationship between altruistic personality and ostracism. Targets with lower altruism are more likely to suffer from ostracism because of the source's inference that these targets have lower social responsibility.

### Moderating Role of Empathy

Empathy refers to the ability of individuals to perceive, understand, and experience other individuals' mental states and emotions and respond to their inner emotions (Davis, 1983; Bernhardt and Singer, 2007; Singer and Lamm, 2010). Studies have shown that empathy can induce individuals to reduce other's pain and urge them to engage in beneficial behaviors. Individuals with high empathy levels are more likely to display explicit prosocial behavior (Yu and Liu, 2006) or show an implicit tendency to help others (Cheng and Yang, 2009). Individuals with high trait empathy are more likely to respond empathetically and experience and understand others' emotional states and cognitive perspectives (Li et al., 2017). Schimel et al. (2006) found that individuals with high trait empathy were more likely to forgive those who misbehaved (such as violent hockey players).

In addition, many studies have shown that empathy has an inhibitory effect on negative behavior. Miller and Eisenberg (1988) found through meta-analysis that there is a significant negative correlation between individual self-reported empathy and attack, and this relationship exists in different age groups and types of attack. Children with high empathy levels tend to show more prosocial behavior and lesser aggression or withdrawal (Sutton et al., 1999). Some Chinese researchers have suggested that empathy can effectively inhibit children's aggressive behavior and that empathy training can reduce their aggressive behavior (Ren, 2002; Wei and Yue, 2002). Chen et al. (2011) also believed that empathy can inhibit aggression and that it is beneficial to the mental health of college students. According to the above empirical evidence, empathy inhibits negative behavior. Ostracism not only harms those who are targeted but also negatively influences the source of ostracism (Poulsen and Kashy, 2012; Nezlek et al., 2015). Therefore, we propose:

**Hypothesis 3:** Empathy plays a moderating role in the relationship between altruistic personality and ostracism. Compared with individuals with low empathy levels, individuals with high empathy levels reduce their ostracism even in the face of low-altruistic goals.

As mentioned earlier, empathy can promote prosocial behavior and inhibit negative behavior; individuals with high empathy levels are more likely to experience and understand others' mental states and emotions (Chen et al., 2011; Li et al., 2017). Empathy might also play a moderating role in the relationship between altruism and ostracism through an indirect path. However, altruism and social responsibility in this study are both traits of ostracism targets. The source of ostracism infers the social responsibility of the target individual according to the individual's altruistic level, and both are relatively stable intrinsic traits of the same individual. Therefore, it is illogical to say that the source of ostracism, that is, individuals with high empathy think that those who are altruistic have more social responsibility and thus reduce ostracism. Therefore, we speculate that the moderating effect of empathy may occur in the second stage, that is, the influence of social responsibility on ostracism—and propose:

**Hypothesis 4:** Empathy plays a moderating role in the mediating effect of social responsibility. Compared with individuals with low empathy levels, individuals with high empathy levels will not increase their ostracism intention even if they realize that the social responsibility of target individuals with low altruism is lower.

## Research Overview

We hypothesized that individuals with low scores in altruism may be more prone to ostracism and that social responsibility would account for this effect. In Study 1, we expected that ostracism would be associated with lower altruism levels. In Study 2, we further validated the causal relationship between altruism and ostracism by laboratory experiments and tested the moderating role of empathy on the relationship between altruism and ostracism. Based on Study 2, Study 3 set the experimental situations as high-altruistic and low-altruistic situations to manipulate the altruistic level of the targets and continued to explore the participants' ostracism intentions to the targets. At the same time, it also examined the mediating role of social responsibility and whether empathy played a moderating role in the mediating effect of social responsibility.

### Study 1

The goal of Study 1 was to examine the relationship between altruistic personality traits and chronic ostracism.

### Method

Study 1 was an online survey. We used the Questionnaire Star platform to compile the questionnaire and generate a link and then posted the link to the WeChat group to recruit college students to participate in the current study. Participants were invited to complete the online questionnaire, including social ostracism, altruistic personality traits, and demographic variables. The measures were presented randomly. Data from 360 participants (93 male and 267 female students, mean age 21.89 ± 2.91 years) were used in Study 1.

Participants reported their altruistic personality traits by completing the 17-item Self-report Altruism Scale (e.g., “I have delayed an elevator and held the door open for a stranger,” and “I have given directions to a stranger;” Rushton et al., 1981; Tang et al., 2015; α = 0.80). Ratings were made on a scale from 1 (never) to 5 (always). The higher the participants' score on the scale, the higher their level of altruistic personality. Participants also reported their experiences of ostracism by completing the 11-item Ostracism Experience Scale (e.g., “In general, others treat me as if I am invisible,” and “In general, others look through me as if I do not exist;” Gilman et al., 2013; α = 0.83). Zhang et al. (2018) verified the good reliability and validity of this scale among Chinese adolescents. Ratings were made on a scale from 1 (hardly ever) to 5 (almost always), with six items scored in reverse. In the reverse scoring, the higher the score, the higher the level of being ostracized^1^.

Since all variables in this study were measured using a questionnaire, the common method bias was tested according to the Harman's single factor test. All variables, such as altruism and ostracism, were combined for exploratory factor analysis, and principal component analysis was used to examine the results of factor analysis before rotation. According to the hypothesis of the Harman's single factor test, if there is only one factor extracted or one of these factors extracted explains most of the variation, a serious common method bias may be considered. Our results showed that there were seven factors with eigenvalues greater than 1 when not rotated, and the variance interpretation rate of the first factor was 18.69%, which was lower than the standard of 40%, indicating that there was no serious common method bias in this study (Zhou and Long, 2004), and the relationship between the variables was credible.

### Results and Discussion

Means and standard deviations of variables such as altruism (*M* = 3.12, *SD* = 0.52) and ostracism (*M* = 2.49, *SD* = 0.62) were calculated, and Pearson correlation analysis was conducted. Results showed that altruism was negatively correlated with ostracism (*r* = −0.32, *p* < 0.01).

Results revealed that there was a significant negative correlation between altruistic personality traits and ostracism, which is consistent with hypothesis 1 of the present study. Because of the cross-sectional nature of the data collected by the questionnaire, this study cannot fully determine the causal relationship of individuals' altruistic personality traits and their being ostracized in daily life. Therefore, Study 2 was conducted to further examine the relationship of the two using a laboratory experiment method to manipulate the targets' altruistic personality traits. Furthermore, previous studies indicated that individuals with high levels of trait empathy were more likely to experience others' state of affective and cognitive perspectives (Li et al., 2017); therefore, the effects of empathy of participants on the relationship between altruism and ostracism was examined in Study 2.

## Study 2

The goal of Study 2 was to experimentally examine the association between altruistic personality and ostracism and the moderating role of empathy.

### Method

#### Participants

A total of 93 college students (22 men and 71 women) participated in this experiment, with an average age of 20.85 ± 2.63 years. Participants that were recruited from the WeChat groups were randomly assigned to two situations: a high-altruistic target and a low-altruistic target, and were informed that they would complete an experiment on the impression of others individually in the behavioral laboratory.

#### Procedure

First, the participants completed the Interpersonal Reactivity Index-C (IRI-C) revised by Zhang et al. (2010). This scale is a 22-item self-report measure designed to assess participants' trait empathy (e.g., “For those less fortunate than I am, I often have a soft and caring feeling,” and “Sometimes I imagine how things look like from my friends' point of view to understand them better;” α = 0.79), and ratings were made on a scale from 0 (totally inconsistent) to 4 (totally consistent). The higher the participants' score on the scale, the higher their level of trait empathy.

Then, the participants were presented with a text material with two paragraphs (about 250 words), which described the target named Mason, a sophomore in the school of information. Simultaneously, they were instructed to imagine what kind of person Mason was and how long they were willing to engage with Mason in their mind. Participants in high-altruistic target situations read the following description of Mason's personality traits: “In general, Mason was generous, caring, helpful, and considerate,” whereas participants in the low-altruistic target situation read the following description of Mason's personality traits: “In general, Mason was a mean, uncaring, unhelpful, and inconsiderate person.” These descriptions of Mason's personality traits were compiled based on previous researchers' definition of altruistic personality (Rushton et al., 1981).

Next, the participants needed to complete a manipulation check of the target's personality traits to test whether there was any difference in the target's altruistic personality that they perceived. Three items, which ostensibly seemed to be reading comprehension questions, were used to conduct a manipulation check. Two of the three items were unrelated to the purpose of the study, and the item describing Mason's personality traits was the real item of manipulation check: “Mason was generous, caring, helpful, and considerate,” and was rated on a 5-point scale.

Subsequently, ostracism intention was measured using the 7-item Ostracism Intention Scale (e.g., “I could ignore Mason,” and “I may give Mason a silent treatment;” α = 0.85), which has been used in the research of agreeableness (Hales et al., 2016). Participants were asked to imagine that Mason joined a students' association that they were in and how they would treat Mason. The likelihood of engaging in behaviors such as excluding, ignoring, and giving silent treatment toward Mason was assessed by completing the scale. Ratings were made on a scale from 1 (completely disagree) to 5 (completely agree). The higher the participants' score on the scale, the higher the intention to ostracize the target.

### Results

#### Manipulation Checks

The independent sample t-test was conducted with different experimental situations as independent variables and participants' altruistic ratings of the target as dependent variables. Results showed that participants' altruistic ratings of the target in the high-altruistic situation (*M* = 3.48, *SD* = 1.20) were significantly higher than those in the low-altruistic condition [(*M* = 1.26, *SD* = 0.63): *t*(91) = −9.68, *p* < 0.001, *d* = −2.13, 95% CI = −2.68, −1.77], indicating that altruistic manipulation was effective.

##### Preliminary Analyses

Means and standard deviations of the main variables are presented in Table 1. As expected, altruism was negatively correlated with ostracism.

**Table 1:** Means and standard deviations of the variables in study 2.

	***M***	***SD***	**1**	**2**	**3**	**4**
1. Gender	–	–	1			
2. Age	20.85	2.63	−0.32^**^	1		
3. Altruism	1.67	0.47	−0.07	0.18	1	
4. Ostracism	1.96	0.76	0.01	−0.15	−0.60^**^	1
5. Empathy	2.49	0.62	0.14	−0.01	−0.05	−0.03

** *p < 0.01. Gender was coded such that 1, male and 2, female*.

##### Effect of Altruism on Ostracism

The independent sample *t*-test was conducted with different experimental situations as independent variables and participants' ostracism intention toward the target as dependent variables. Results showed that participants' ostracism intention toward the target in the low-altruistic situation (*M* = 2.60, *SD* = 0.84) was significantly higher than that in the high-altruistic condition [(*M* = 1.63, *SD* = 0.46): *t*(91) = 7.19, *p* < 0.001, *d* = 1.58, 95% (CI) = 0.70, 1.23].

##### Moderating Role of Empathy

According to the moderation effect test proposed by Wen and Ye (2014), examining the moderating role of empathy in the relationship between altruism and ostracism. In order to minimize the loss of data, we set the data with scores in the lower 50% as the low empathy group, and the data with scores in the higher 50% as the high empathy group based on the mean value of empathy. A 2 (the high-altruistic target vs. low-altruistic target) ×2 (high in empathy vs. low in empathy) analysis of variance showed that the interaction terms of altruism and empathy did not significantly predict the ostracism intention: *F*_(1, 92)_ = 0.13, *p* = 0.72. That is, the relationship between the target's altruism and participants' ostracism intention toward the target was not moderated by empathy level.

### Discussion

By manipulating the altruistic level of the target through laboratory experiments, this study investigated participants' ostracism intention toward the target with different altruism levels and tested the moderating role of participants' trait empathy on the relationship between the two. It was found that participants' ostracism intention toward the target in the low-altruistic situation was significantly higher than that in the high-altruistic condition, which confirmed the causal relationship between altruism and ostracism and further proved Hypothesis 1, that is, the target's altruism personality trait is a cause of its being ostracized.

However, the moderating role of empathy on the relationship between altruism and ostracism was not significant, which is inconsistent with Hypothesis 3. As mentioned above, empathy promotes prosocial behaviors and inhibits negative behaviors (Yu and Liu, 2006; Chen et al., 2011). Therefore, we hypothesized that although empathy on the relationship between altruism and ostracism has no direct moderating role, it may also act through an indirect path; thus, we further explored the moderating role of empathy in Study 3.

## Study 3

Study 3 identified the causal relationship between altruism personality traits and ostracism. This study further examined the mediating role of social responsibility between altruism and ostracism and the moderating role of empathy on the mediating effect of social responsibility, that is, a moderated mediation model.

### Method

#### Participants

Participants that were recruited from the WeChat groups were randomly assigned to two situations, a high-altruistic target and a low-altruistic target, and were invited to complete an experiment on our impression of others in the behavioral laboratory. A total of 129 college students (32 men and 97 women) participated individually in this experiment, with an average age of 20.81 ± 2.50 years.

#### Materials and Procedure

The procedure of Study 3 is basically the same as that of Study 2, except that it increased the measurement of social responsibility. First, participants completed the Interpersonal Reactivity Index-C (Zhang et al., 2010; α = 0.79), which is the same as in Study 2. After reading a text material with two paragraphs (about 250 words, same as Study 2), participants also needed to complete a manipulation check of the target's personality traits (same as Study 2).

Participants then completed the Adolescent Students' Responsibility Questionnaire compiled by Cheng (2002) to report their evaluation of the target's social responsibility. There were 23 items on the scale and rated on a 5-point scale. To make the items more suitable for the study, we modified the expression of items. For example, “I can maintain the collective code of conduct” was adjusted to “I think Mason can maintain the collective code of conduct,” and “I will stop it when I see others littering” was adjusted to “I think Mason will stop it when he sees others littering;” (α = 0.94). The higher the participants' score on this scale, the higher the targets' social responsibility.

Finally, participants completed the measurement of the ostracism intention (same as Study 2; α = 0.88).

### Results

#### Manipulation Checks

The independent sample t-test was conducted with different experimental situations as independent variables and participants' altruistic ratings of the target as dependent variables. Results showed that participants' altruistic ratings of the target in the low-altruistic situation (*M* = 1.27, *SD* = 0.65) were significantly lower than those in the high-altruistic condition [(*M* = 3.49, *SD* = 1.21): *t*(127) = −12.97, *p* < 0.001, *d* = −2.28, 95% CI = −2.57, −1.89], indicating that altruistic manipulation was effective.

#### Preliminary Analyses

Means and standard deviations of the main variables were calculated, and Pearson correlation analysis was conducted. As depicted in Table 2, results showed that altruism was negatively correlated with ostracism, altruism was positively correlated with social responsibility, and social responsibility was negatively correlated with ostracism.

**Table 2:** Means and standard deviations of the variables in study 3.

	***M***	***SD***	**1**	**2**	**3**	**4**	**5**
1. Gender	1.75	0.43	1				
2. Age	20.81	2.50	−0.27^**^	1			
3. Altruism	1.50	0.50	0.04	0.04	1		
4. Social responsibility	2.75	0.70	−0.01	0.07	0.65^**^	1	
5. Ostracism	2.26	0.88	−0.11	−0.07	−0.50^**^	−0.55^**^	1
6. Empathy	2.62	0.43	0.05	0.09	0.03	0.08	−0.17

** *p < 0.01. Gender was coded such that 1, male and 2, female*.

##### Effect of Altruism on Ostracism

The independent sample t-test was conducted with different experimental situations as independent variables and participants' ostracism intention toward the targets as dependent variables. The results showed that participants' ostracism intention toward the targets in the low-altruistic situation (*M* = 2.70, *SD* = 0.85) was significantly higher than that in the high-altruistic condition [(*M* = 1.82, *SD* = 0.67): *t*(127) = 6.57, *d* = 1.16, *p* < 0.001, 95% CI = 0.62, 1.15].

##### Mediating Role of Social Responsibility

**Figure 1:**
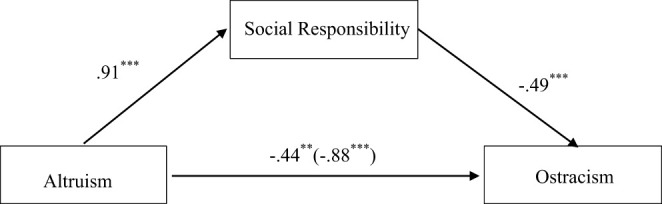
Mediating role of social responsibility in study 3. *Note*: **p < 0.01; ***p < 0.001.

According to the mediating effect test method proposed by Wen and Ye (2014) and the SPSS macro program PROCESS (Hayes, 2013), bias-corrected bootstrapping analysis with 5,000 samples were used to examine the mediating role of social responsibility. Results revealed that the targets' altruism significantly predicted its social responsibility and participants' ostracism intention toward the targets. When both altruism and social responsibility entered into the regression equation, social responsibility could significantly negatively predict participants' ostracism intention toward the targets; and the 95% confidence interval (−0.72, −0.25) of mediating effect did not include 0, suggesting that social responsibility played a significant mediating role in the relationship between altruism and ostracism. The mediating effect size was −0.44. After controlling for social responsibility, the effect of altruism on ostracism was still significant. Therefore, social responsibility played a partial mediating role in the relationship between altruism and ostracism, and the mediating effect accounted for 50.36% of the total effect. Figure 1 shows the mediation model.

##### Moderating Role of Empathy

According to the test method proposed by Wen and Ye (2014) and the SPSS macro program PROCESS (Hayes, 2013), we examined the moderated mediation model.

First, we examined whether the direct effect of altruism on ostracism was moderated by empathy. The regression equation tested was Y = c_0_ + c_1_ altruism + c_2_ empathy + c_3_ altruism × empathy + e_1_ (equation 1). Results showed that the interaction between altruism and empathy had no significant effect on ostracism intention [β = 0.07, *F*_(1, 125)_ = 0.04, 95% CI = −0.60, 0.74, *p* = 0.84]; that is, the direct effect of altruism on ostracism intention was not moderated by empathy.

According to Wen and Ye (2014), the following analysis could be continued even if the direct effect of the independent variable on the dependent variable was not moderated. Therefore, we examined whether the mediating effect of social responsibility was moderated by empathy. According to the previous hypothesis, empathy only in the second stage played a moderating role; therefore, the regression equation tested was social responsibility = a_0_ + a_1_ altruism + e_2_ (equation 2) and Y = c_0_' + c_1_' altruism + c_2_' empathy + b_1_ social responsibility + b_2_ social responsibility × empathy + e_3_ (equation 3). The results of equation 2 showed that the target's altruistic personality significantly predicted its social responsibility [β = 0.91, *F*_(1, 127)_ = 92.08, 95% CI 0.72, 1.10, *p* < 0.001]. However, the results of equation 3 indicated that the interaction term of empathy and social responsibility had no significant effect on the ostracism intention [β = 0.02, *F*_(4, 124)_ = 17.41, 95% CI −0.42, 0.45, *p* = 0.94].

Therefore, the moderated mediation model had not been verified in our study.

### Discussion

Study 3 found that the altruism level of the target could significantly predict participants' ostracism intention toward the target, suggesting that participants' ostracism intention toward the target in the low-altruistic situation was significantly higher than that in the high-altruistic condition, which further confirmed hypothesis 1.

Using the measurement of social responsibility, Study 3 also found that social responsibility played a partial mediating role in the relationship between altruism and ostracism intention, which indicated that individuals with low altruism level were more vulnerable to being ostracized, owing largely to the sources' inference of the low social responsibility of the target, which was consistent with hypothesis 2.

In addition, Study 3 examined the moderating effect of empathy. Similar to the results of Study 2, empathy did not moderate the direct effect of altruism on ostracism. Moreover, we also tested the moderating role of empathy on the mediating effect of social responsibility and found that there was still no significant moderating effect of empathy. Therefore, hypothesis 4 was not verified.

## General Discussion

Study 1 investigated individuals' altruistic personality traits and their experience of being ostracized in daily life through questionnaires and found that there was a significant negative correlation between altruism and ostracism. Studies 2 and 3 manipulated the targets' altruism level in laboratory experiments, asked the participants to evaluate their ostracism intention toward the targets, and further verified the hypothesis: “individuals' level of altruism is a cause of their being ostracized.”

The results of the present study are consistent with those of previous studies. Kurzban and Leary (2001) argue that ostracism has an evolutionary function for the survival and development of groups; ostracism can protect other group members from exploitation by expulsing those who are burdensome, harmful, and diseased, thereby enhancing the group's adaptability. Therefore, groups will prefer cooperative individuals and reject those who add burden to the group (Wesselmann et al., 2015). However, individuals with low altruism tend to be selfish and do not pay attention to others' needs and interests (Luo and Dai, 2015), which may threaten the survival and development of the group. This may not be conducive in enhancing the adaptability of the group and thus more prone to ostracism.

In addition, studies have shown that the development of the group calls for joint efforts of group members. If some of the group members engage in free-riding behavior, the enthusiasm and productivity of other members will be seriously affected, the overall benefit of the group will eventually be impaired, and other members bear the corresponding consequences (Jin, 2005). Individuals are naturally highly concerned with fairness, reciprocity, and freedom from exploitation (Graham et al., 2009), and Hales et al. (2016) found that individuals who pay special attention to the principle of fairness showed a stronger tendency to reject individuals with low agreeableness. A study by Rudert et al. (2017) also showed that individuals found it more acceptable to reject those who are indifferent and incompetent because indifferent and incompetent individuals are generally regarded as expendable, whether for a specific group or for the whole society. Individuals with low altruism tend to be more selfish, indifferent, self-centered, and not consider others' interests and needs (Luo and Dai, 2015). They may have fewer knowledge-sharing behaviors in the group and larger free-riding tendency (Constant et al., 1994), which has a negative influence on the development of group and also causes damage to others' interests, thus more vulnerable to be ostracized.

Study 3 explored the mediating role of social responsibility. Results suggested that social responsibility played a partial mediating role in the relationship between altruism and ostracism. The altruistic personality of the target leading to its being ostracized is really due to a low level of social responsibility inference toward the target. On the one hand, our finding on the mediating role of social responsibility is consistent with previous study results (Maruyama et al., 1982; Bierhoff et al., 1991; Egan, 2009; Kerber, 2010; Lee et al., 2011; Yan, 2011; Fan, 2017); on the other hand, the result also indicates that there is a dual path in the effect of altruism on ostracism. Altruism not only directly affects ostracism but also exerts an influence through the path of “altruism–social responsibility–ostracism.” This not only proves that individuals' low altruism is an important cause of being socially ostracized but also suggests that the sources' evaluation of the target's social responsibility is an important internal psychological process in making ostracism decisions.

Previous studies on the relationship between personality traits and ostracism have also examined the internal psychological mechanism. For example, Hales et al. (2016) believed that the reason why low-agreeableness targets were more likely to suffer from ostracism was that they were not trusted. Feinberg et al. (2014) also explored why selfish individuals are more likely to be rejected in the workplace context. They found that selfish individuals' performance in the last round of task would bring them poor reputation and thus lead them to be more excluded in the next round of task, that is, reputation played a mediating role between selfishness and rejection. Although the present and previous studies examined different variables when exploring the internal mechanism, the choice of these variables is based on views of the evolutionary function of ostracism (Kurzban and Leary, 2001). Ostracizing members that could threaten the survival and development of the group is helpful to improve the adaptability of the group and can also save other members in the group from being exploited.

Studies 2 and 3 explored the moderating effect of empathy and found that empathy had no significant moderating effect on the direct effect of altruism on ostracism intentions, nor did the indirect effect of the two, which is inconsistent with previous studies. Previous studies have shown that empathy could not only promote implicit helpful tendencies (Cheng and Yang, 2009) or explicit prosocial behaviors (Yu and Liu, 2006) but also inhibit negative behaviors (Miller and Eisenberg, 1988; Chen et al., 2011). Individuals with high trait empathy are more likely to experience and understand others' emotional states and cognitive views (Li et al., 2017). In the present study, ostracism is a negative behavior; reducing ostracism of the target is also a prosocial behavior to some extent. According to previous studies, empathy should have been able to moderate the relationship between altruism and ostracism. However, results of this study showed that the moderating effect of empathy was not significant.

Although this result is inconsistent with our hypothesis, in fact, it is not incomprehensible. According to the evolutionary function of ostracism, groups will prefer cooperative individuals and reject those who may threaten the survival and development of the group to enhance its adaptability (Kurzban and Leary, 2001; Wesselmann et al., 2015). Individuals with low altruism tend to be selfish and do not pay attention to the interests of others; such individuals have difficulty making contributions to the group and are easy to be free riders, thus damaging the interests of other members (Constant et al., 1994), which is unfair. Fair, however, is an important pursuit in individuals' lives (Wu and Zhou, 2012), and individuals are naturally and highly concerned with fairness, mutual benefit, and freedom from exploitation (Graham et al., 2009). They are not only concerned with their own pay and reward but also with others' (Tabibnia et al., 2008) and form a feeling of fairness by comparison (Weng, 1999). Therefore, empathy had no significant moderating effect on the relationship between altruism and ostracism, which may be due to the fact that low altruism is unfavorable to group development and unfair to other members of the group.

In addition, emotional sharing theory holds that emotional sharing between individuals is the basis of empathy (Jeannerod, 1999; Decety and Sommerville, 2003), whereas emotional sharing is largely dependent on the automatic association between the expression of others' emotions and emotional experience one has experienced (Decety and Lamm, 2006). In this study, the target the participants faced was a virtual character in the reading materials rather than a real individual face to face. The participants were not aware of the emotional reactions of the target and could not achieve emotional sharing. Therefore, in this non-real interaction situation, it is difficult for individuals with high trait empathy to experience the targets' pain of ostracism; therefore, the moderating effect of empathy on the relationship between altruism and ostracism is not significant. It has been shown that even when observing others suffering from ostracism, individuals will also experience pain similar to that of being excluded (Giesen and Echterhoff, 2017). Therefore, we suggest future research on the cause of ostracism to further optimize experimental situations and set up experiments with higher ecological validity to examine whether individuals will really reduce their ostracism level when they can very clearly experience the pain of the targets.

Through a questionnaire combined with laboratory experiments, this study systematically explored the relationship between altruism and ostracism and broke the previous “results–intervention” research mode. This study helped increase the literature on the causes of ostracism by deeply understanding why social ostracism occurred and wished to enlighten future researchers in this field to follow the complete “reason–results–intervention” research model (Zhang et al., 2018).

Ostracism occurs in interpersonal interaction, and its causes involve many aspects such as the target, source, and situation. This study not only examined the target with the kind of personality traits that are likely to be ostracized from the target's perspective but also investigated the kind of personality traits that could lower its ostracism intention from the source perspective, combining the two angles of the target and source to fully understand the emergence of ostracism, which makes the results closer to the real process of interpersonal interaction.

Our study has limitations, which we believe can serve as avenues for future related investigations. We only explored the altruism personality trait, for the rest of the Big Five lack of exploration; therefore, we cannot be sure what personality traits and which combination of characteristics will lead individuals to be more likely to suffer ostracism. Future researchers can further explore the effect of other personality traits on ostracism.

In addition, in Studies 2 and 3, we only manipulated the altruism level of the target but did not measure the altruism level of the participants (the source); therefore, the interaction between the target's altruistic personality traits and participants' altruism could not be tested. According to the similarity law of interpersonal attraction, similar personality characteristics increase liking and attraction (Boyden et al., 1984; Gonzaga et al., 2007). If the target's level of altruism is exactly similar to that of the participants, then participants' ostracism intention toward the target may also be low even if the target's level of altruism is low. Therefore, future researchers should simultaneously investigate whether the similarity or difference in the same personality traits between the target and source will have an interactive impact on ostracism.

Furthermore, the altruism investigated in this study belongs to the personality trait in the Big Five, which is studied under the framework of the western personality model. However, according to the behavior classification hypothesis of Wang and Cui (2006), cross-cultural differences in personality structure are not only reflected by different personality dimensions but also by differences in the connotation of each dimension, and they believe that the Big Seven factor model can more accurately reflect the personality characteristics of Chinese people. In addition, the “differential pattern” of interpersonal relationship in Chinese culture is very evident (Yang et al., 2008), which may make Chinese people consider more about their identity when excluding or accepting someone and less about their personality. Therefore, future researchers can adopt the Big Seven factor model to conduct local research and investigate the influence of identity characteristics of the target and source and whether they belong to the same group on the ostracism intention.

Besides, we acknowledge the limitation of the approach taken, namely, all the outcome data was self-report, which may easily increase the risk of common method bias, especially the social desirability bias. Although we have conducted a Harmon one-factor test and the results have showed no serious common method bias in our study, it is better for future studies to adopt more diversified methods in the research design and measurement process to prevent the risk of common method bias, for instance, protecting the anonymity of participants, measuring variables from different sources and time points, reducing the predictability of the research objective, balancing the order effect of items and so on (Zhou and Long, 2004).

Finally, the statistical power of Studies 2 and 3 should be taken cautiously given that the sample size were relatively small in Studies 2 and 3 for exploring the moderated mediation model.

## Data Availability Statement

The raw data supporting the conclusions of this article will be made available by the authors, without undue reservation.

## Ethics Statement

The studies involving human participants were reviewed and approved by the Research Ethics Committee of Renmin University of China. The patients/participants provided their written informed consent to participate in this study.

## Author Contributions

LH contributed to the conception and design. LH and YZ contributed to the collection, analysis, and interpretation of data. DZ and YZ contributed to drafting the article. DZ contributed to revising the article critically. All authors contributed to the article and approved the submitted version.

## Conflict of Interest

The authors declare that the research was conducted in the absence of any commercial or financial relationships that could be construed as a potential conflict of interest.

## Footnotes

^1^Although the scale includes two dimensions, researchers generally use a total score to measure the degree of ostracism experienced by individuals (e.g., Hales et al., 2016).
